# Plastic straw: future of high-speed signaling

**DOI:** 10.1038/srep16062

**Published:** 2015-11-03

**Authors:** Ha Il Song, Huxian Jin, Hyeon-Min Bae

**Affiliations:** 1 Korea Advanced Institute of Science and Technology (KAIST), Department of Electrical Engineering, Daejeon, 305-701, Republic of Korea

## Abstract

The ever-increasing demand for bandwidth triggered by mobile and video Internet traffic requires advanced interconnect solutions satisfying functional and economic constraints. A new interconnect called E-TUBE is proposed as a cost-and-power-effective all-electrical-domain wideband waveguide solution for high-speed high-volume short-reach communication links. The E-TUBE achieves an unprecedented level of performance in terms of bandwidth-per-carrier frequency, power, and density without requiring a precision manufacturing process unlike conventional optical/waveguide solutions. The E-TUBE exhibits a frequency-independent loss-profile of 4 dB/m and has nearly 20-GHz bandwidth over the V band. A single-sideband signal transmission enabled by the inherent frequency response of the E-TUBE renders two-times data throughput without any physical overhead compared to conventional radio frequency communication technologies. This new interconnect scheme would be attractive to parties interested in high throughput links, including but not limited to, 100/400 Gbps chip-to-chip communications.

With the exponential growth of network traffic, the throughput of data centres is approaching the zettabyte regime^1^. Such trends drive a rapid increase in the input/output (I/O) bus speed along with the advancement of integrated circuit (IC) technologies^2^,^3^. For decades, conductor-based electrical interconnects have been widely adopted for various wireline communication systems owing to the interconnect’s cost/power efficiencies. However, the skin effect, caused by electromagnetic induction, imposes a fundamental limitation on the channel bandwidth, which in turn limiting the throughput-distance product to 100 Gbps∙m. As such, it has been generally believed that the optical interconnects will eventually replace the high-speed electrical interconnects in the foreseeable future^4^,^5^,^6^,^7^,^8^,^9^. Nevertheless, the widespread use of optical interconnects is severely challenged by the overwhelming replacement cost^10^,^11^,^12^,^13^. The high-precision manufacturing required for the electrical-to-optical (EO) and optical-to-electrical (OE) conversion process places a significant economic barrier in satisfying the market demand: <10 ¢/pin. Therefore, in this paper, a cost-and-power-effective all-electrical-domain broadband dielectric waveguide solution referred to as an E-TUBE is proposed to replace conventional conductor-based interconnects in high-speed, short-reach communication links.

The E-TUBE is a dielectric waveguide clad with a thin metal layer, which achieves nearly 20-GHz bandwidth over the V band (~70 GHz) and exhibits a frequency-independent insertion loss of 4 dB/m. The frequency-independent loss-profile of the E-TUBE does not require complex equalizers commonly used in conductor-based interconnects, which lowers the power budget of an overall link.

A metal waveguide has been used to transfer microwaves with high energy efficiency. However, due to its high rigidity, a hollow metal pipe creating total reflection at the metal boundaries is expensive and inconvenient. In contrast, a dielectric waveguide, although flexible and easy to use^14^, it has not been considered as an alternative for the conductor based interconnects due to 1) poor manufacturability^15^,^16^ and 2) low power efficiency^17^ caused by limited bandwidth-to-centre frequency ratio and 3) intrinsic energy loss due to the field leakage and high bending loss caused by refraction occurring at the boundaries of the bended dielectric waveguide.

The E-TUBE is a metal clad dielectric waveguide, which holds the advantages of both metal and dielectric waveguides. As compared with the metal waveguide, the manufacturability of the E-TUBE is improved significantly by the replacement of the hollow space with the flexible dielectric tube, thereby enabling a simple foil wrapping for the metal cladding. In addition, the cross-section of the waveguide is reduced by the increase in the dielectric constant. As compared with the dielectric waveguide, the metal cladding of the E-TUBE prevents electromagnetic leakage and enables low bending loss, thanks to its high confinement. The E-TUBE overcomes aforementioned functional limitations while using only standard manufacturing processes. In addition, the resonator modes within the waveguide are controlled to further improve signal integrity. A single-sideband signal transmission enabled by the inherent frequency response of the E-TUBE achieves two-times data throughput without any physical overhead when compared with conventional radio frequency (RF) communication technologies.

## Results

### Design and modelling of E-TUBE link

**Figure 1:**
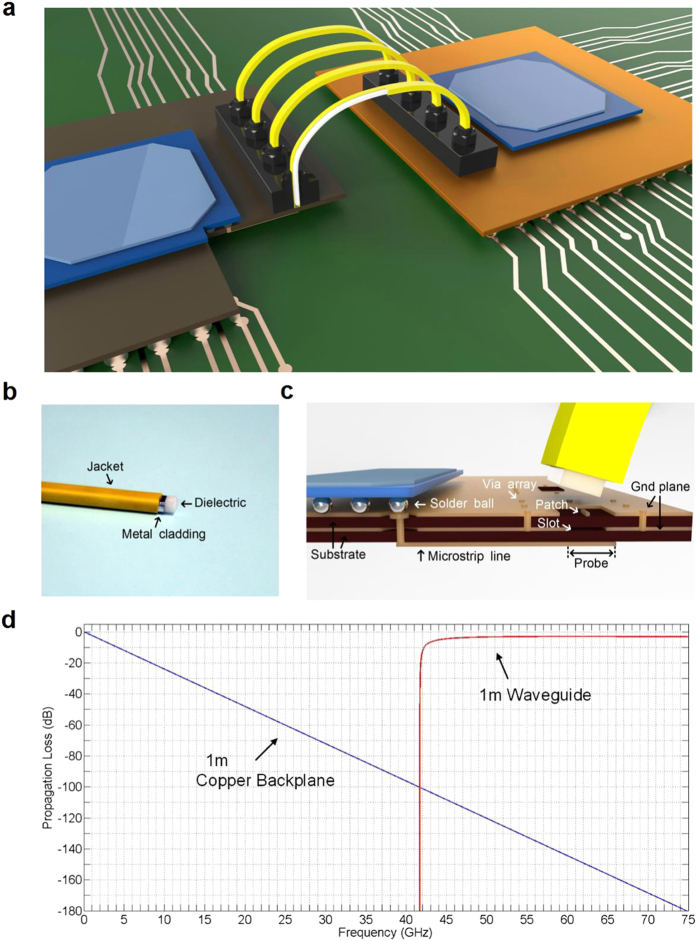
Illustration of E-TUBE links and comparison with electrical conductor links. (**a**) A conceptual illustration of the proposed interconnect. The waveguides and the microstrip circuits for multi-channel communication. (**b**) A photograph of the waveguide. Thin metal cladding covers the dielectric waveguide. A jacket is used for physical robustness of waveguide. (**c**) An illustration of the waveguide and the interface. Structure of the Microstrip-to-Waveguide Transition on the Printed Circuit Board is shown. Waveguide-to-Board connector is omitted. (**d**) Comparison of calculated propagation loss along 1-m copper backplane and 1-m dielectric waveguide with metal cladding.

Figure 1a shows the overall E-TUBE interface. The dielectric waveguide clad with a thin metal layer (Fig. 1b) is plugged directly on the printed circuit board (PCB). The Waveguide-to-Board connector is designed to couple the waveguides vertically to the board, which is crucial for cost-and-area effective throughput increase. The designed waveguide is 4.0-mm wide and 2.0-mm thick, allowing the propagation of the *TE*_10_ mode. The V band RF signal is transferred efficiently from the microstrip line to the waveguide via the transition. The transition is manufactured by using only the conventional PCB lamination process appropriate for low-cost mass-production as shown in Fig. 1c. A patch element implemented by using a microstrip excites the electromagnetic field in the waveguide. The metal cladding surrounding the dielectric prevents electromagnetic leakage and confines the electromagnetic waves in the E-TUBE. Further, it provides sufficient isolation from undesirable external noise sources and prevents coupling between channels spaced 1 mm apart. Thus, in contrast to conventional wireless communication systems, the E-TUBE can share a single carrier frequency for multi-channels, enabling an area-efficient transceiver design.

Among all the benefits of the E-TUBE, the most important property of the E-TUBE is that the propagation loss of the E-TUBE is frequency independent, as compared to the conductor-based counterparts over millimeter wave frequencies (Fig. 1d). When an alternating electric field is applied, free electrons in a conductor are forced to the outer shell of the conductor and eventually results in skin loss. On the other hand, a dielectric, whose electrons are bounded in the valence band, is polarized and the direction of polarization rotates with respect to the alternating electric field. The dielectric loss that occurs during the process of polarization is determined by the relaxation time of that process^18^. Because the target reach of the E-TUBE is a few meters, a polyethylene foam demonstrating instantaneous polarizability is used to suppress the dielectric loss 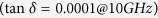 over the passband. As a result, frequency-dependent phenomena such as skin and dielectric losses rarely occur in the E-TUBE over the target frequency range. This frequency-independent loss-profile of the E-TUBE enables high-speed data transmission without any equalization, which is critical for low-power high-speed I/O design.

**Figure 2:**
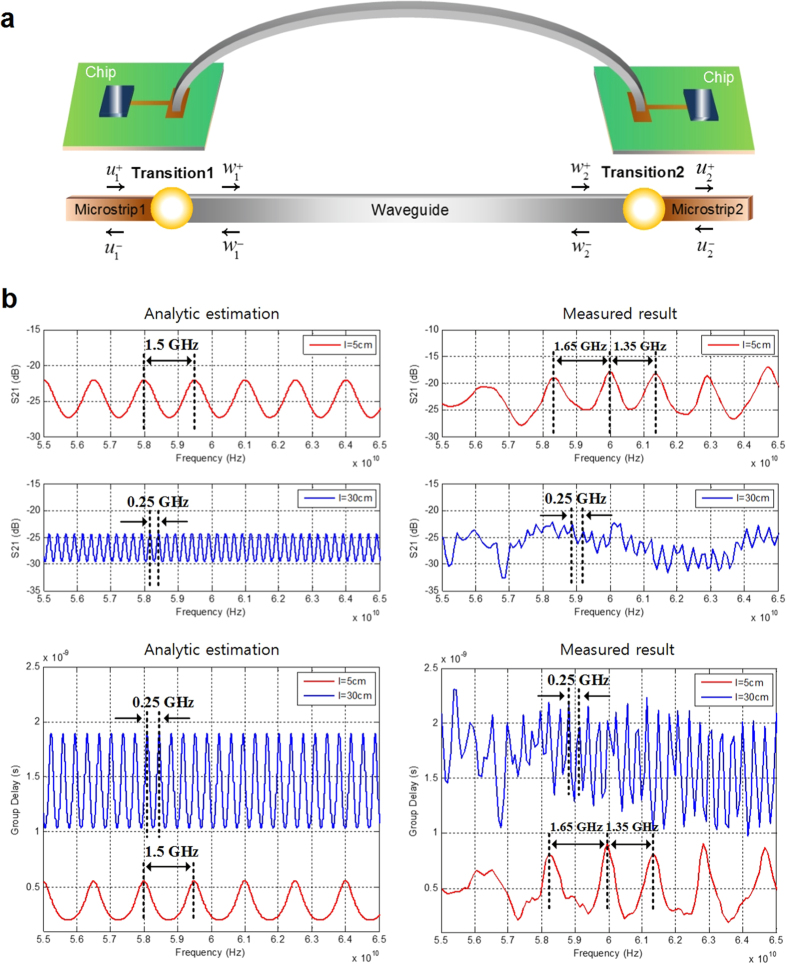
Modelling of E-TUBE link and waveguide-length-dependent frequency-domain ripple. (**a**) A simplified connection diagram of the E-TUBE and an equivalent reciprocal two-port network model with transmitted waves and reflected waves. The incident wave at transition1 in Microstrip1, 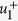, the reflected wave at transition1 in Microstrip1, 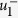, the transmitted wave at transition1 in Waveguide, 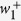, the reflected and re-entered wave at transition1 in Waveguide, 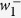, the incident wave at transition2 in Waveguide, 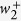, the reflected wave at transition2 in Waveguide, 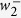, the transmitted wave at transition2 in Microstrip2, 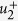, the reflected and re-entered wave at transition2 in Microstrip2, 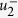. (**b**) Simulated and measured waveguide-length-dependent frequency-domain ripple of 5 cm and 30 cm waveguides in S21 and group delay.

However, low loss along the waveguide constructs resonator modes^19^. The reflected wave created at either ends of the waveguide bounces back and forth within the waveguide, which creates a waveguide-length-dependent frequency-domain ripple. Figure 2a shows the simplified connection diagram of a dielectric interconnect and the equivalent two-port reciprocal network models including the transmitted and reflected waves at each transition. The relations between the transmitted waves and the reflected waves at each transition are described in Supplementary equations (S1) and (S2). The propagating wave along the waveguide can be expressed by Supplementary equation (S3).

Figure 2b shows the simulated results of Supplementary equations (S6) and (S7) under different lengths of the waveguide (e.g., 5 cm and 30 cm). Corresponding measurement results are supplied to validate the modelling of the waveguide-length-dependent frequency-domain ripple. In this simulation, we intentionally created a waveguide model with high reflection at the discontinuities in order to emphasize the importance of careful ripple suppression. The analytic ripple frequency of a 5-cm E-TUBE, 1.5 GHz, matches with the measured average ripple frequencies of 1.65 GHz and 1.35 GHz. The measured ripple frequency of a 30-cm E-TUBE also matches well with the analytic estimation. The waveguide-length-dependent frequency-domain ripple increases with the elongation of the waveguide and eventually causes bit-error-events. Strategies for resolving this problem can be derived from Supplementary equations (S6) and (S7). First, the reflection coefficient at the transition should be made small; next, adequate attenuation should be taken along the waveguide while keeping the channel loss at a low level; finally, an appropriate material with low dielectric constant must be chosen for the waveguide.

Figure 1c shows the slot-coupled Microstrip-to-Waveguide Transition (MWT). The MWT is designed to suppress the reflection occurring at the discontinuities between the waveguide and the microstrip line^20^,^21^,^22^. Slot-embedded geometry offers coupling between the microstrip line and the waveguide by producing a double-tuned resonance, which increases the bandwidth of the transition^23^. The thickness and the dielectric constant of the substrate and the length of the probe element must be chosen to achieve low-quality-factor frequency response to ensure a wide bandwidth. The relations between the quality factor and each parameter are described in Supplementary equations (S18)–(S21): 1) The quality factor decreases in proportion to the resonant frequency. Thus, a higher resonant frequency increases the bandwidth. Considering implementation feasibility, the V band is chosen for achieving a wide bandwidth^24^,^25^,^26^. 2) The quality factor is proportional to the coupling coefficient between the microstrip line and the waveguide. The power delivered to surface wave modes increases in proportion to the electrical thickness of the substrates at millimeter wave frequencies^27^. As the coupling into surface wave modes is a form of loss in the MWT, a thick and high-*ε*_*r*_ substrate lowers the coupling coefficient, eventually increasing the bandwidth. Two core substrates are made of 0.254-mm-thick Rogers RO3010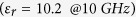. 3) The quality factor is minimized when the length of the probe is equal to half wavelength at resonant frequency. Hence, the length of the probe element should be determined carefully. The selection of a high-*ε*_*r*_ substrate is effective in reducing the dimension of the MWT because the lengths of the probe, slot, and patch can be decreased.

### Measurement of channel frequency response and eye diagram

**Figure 3:**
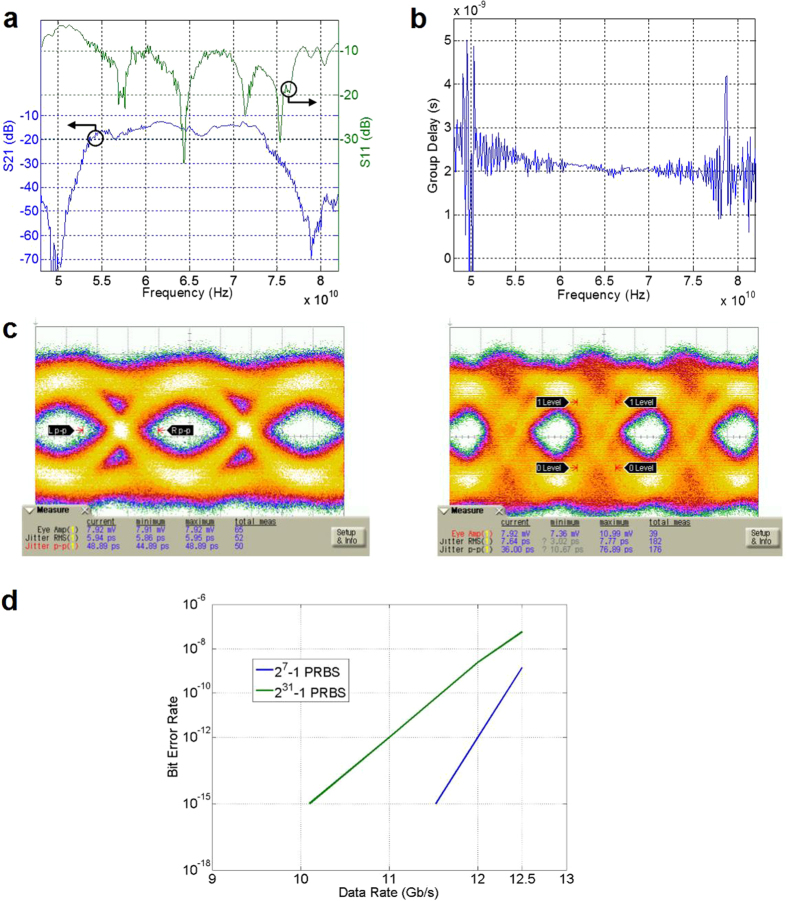
Channel characteristic, eye diagrams and bit-error-rate of single-sideband transmission. (**a**) Measured S-parameter of a 0.5-m E-TUBE channel. (**b**) Measured group delay of a 0.5-m E-TUBE channel. (**c**) The eye diagrams at 12.5 Gb/s and 17 Gb/s with PRBS length of 2^31^–1. (**d**) Bit-error rate measurements with the PRBS patterns of 2^7^–1 and 2^31^–1.

The frequency response of the channel is monitored using S-parameters, and is shown in Fig. 3a. The return loss is under −10 dB in the frequency range of 54.6 GHz to 77.4 GHz. The measured insertion loss is 13 dB at 73 GHz in a 0.5-m channel and increases in proportion to the distance at a rate of 4 dB/m. The group delay, which is determined by the boundary condition between the dielectric waveguide and the cladding, is inversely proportional to frequency (Fig. 3b) and the variation increases in the vicinities of cutoff frequencies.

To mitigate the signal distortion caused by the group delay variation, carrier frequency must be set distant from the low frequency region where group delay varies significantly. The incident RF signal into the E-TUBE is generated by using a conventional double sideband modulation scheme. However, since the upper sideband of the modulated signal is suppressed by the sharp roll-off response of the E-TUBE, only the lower sideband signal is transmitted. Consequently, the data rate can be doubled, as compared to conventional double-sideband transmission. Such an extraordinary sharp cutoff channel characteristic is achieved by optimizing the parameters relevant to coupling between the microstrip line and the waveguide (Supplementary Note 4), and enables bandwidth-efficient data transmission, as compared to ordinary RF communication technologies without any hardware overhead.

Figure 3c shows the eye diagram at 12.5 Gb/s and 17 Gb/s with pseudorandom binary sequence (PRBS). Single-ended PRBS31 with the magnitude of 1.2 V_pp_ was up-converted and down-converted by GaAs Schottky diode-based balanced mixers (SAGE Millimeter Inc.). A 18.325-GHz sinusoidal signal from an Agilent E8257D Signal Generator is multiplied by a factor of four to 73.3 GHz by using a S15 MS WR15 Frequency Extension Module (OML Inc.) to create the carrier signal. Figure 3d shows the bit-error-rate (BER) measurements conducted with a 0.5-m E-TUBE channel. PRBS patterns of 2^7^–1 and 2^31^–1 are applied to the mixer while increasing the data rate. A BER of less than 10^−12^ is observed for data rates less than 12 Gb/s with the PRBS7 pattern. The measured BER at 12.5 Gb/s with the PRBS31 signal is 5.7 × 10^−8^. Suspected causes of BER degradation are: 1) the reflection occurring at the boundary between the RF port of the mixer and the E-TUBE channel, 2) the band limitation of the mixer, 3) the magnitude and group delay variations of the E-TUBE. Tentative equalization schemes for the E-TUBE link is described in Supplementary Note 5.

### Measurement of coupling imperfection and bending radius

**Figure 4:**
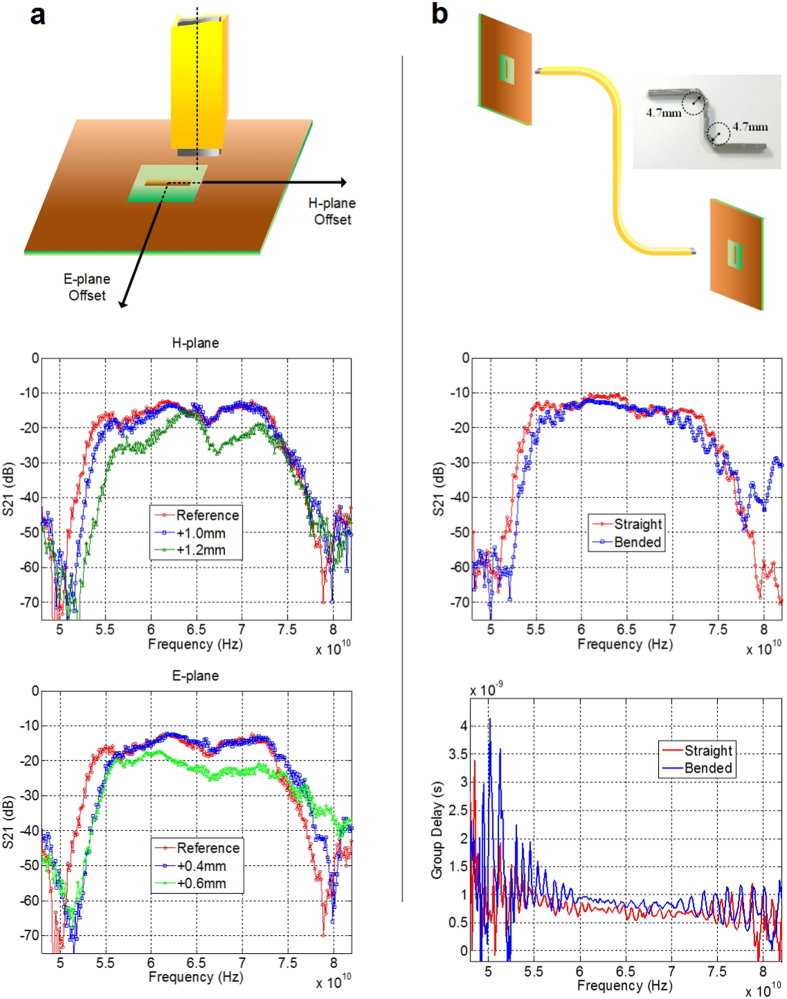
Characterization of coupling imperfection and bending stress (**a**) Vertical and horizontal misalignment of the waveguide. Measured results for coupling imperfection of the waveguide. (**b**) Nonplanar connection: measured results for a 10-cm bended E-TUBE whose bending radius is 4.7 mm and for a 10-cm straight E-TUBE.

Figure 4a shows the measurements with E-plane/H-plane offsets. No significant performance degradation up to 1.2-mm offset in the H-plane (horizontal) and 0.4-mm offset in the E-plane (vertical) are observed. The E-TUBE requires tighter tolerance in the E-plane than in the H-plane, owing to the rectangular shape of the MWT.

As the wavelength of the propagating wave in the E-TUBE is several orders of magnitude longer than that of the optical fibre, coupling imperfections in the order of tens of micrometers do not affect the overall performance of the E-TUBE. This large tolerance enables the usage of low-cost connectors without soldering or pressure contact. Figure 4b shows the nonplanar connection between the transmitter and the receiver. In order to evaluate the effect of bending radius, a bent E-TUBE whose bending radius is 4.7 mm is compared to a straight E-TUBE. As long as the dielectric maintains continuity without a break, the bent E-TUBE shows negligible performance degradation compared with the straight E-TUBE in S_21_ and group delay measurements. The E-TUBE’s weak sensitivity to coupling imperfection and bending stress are critical for its low-cost widespread deployment.

## Discussion

Optical solutions are generally believed to replace high-speed conductor-based interconnects even in short-reach links. However, the widespread use of optical communication systems in high-volume short-reach applications is yet to gain acceptance due to its high-cost components, high-precision manufacturing process requiring micron-level accuracy, and the sheer physical size of modules. Moreover, designs with a practical balance of performance/robustness/power consumption of optical devices erode the potential advantage of the optical fibres. This being the case, the E-TUBE proposes a new interconnect scheme suitable for next-generation high-speed I/O interfaces. The E-TUBE demonstrates an unprecedented level of performance in terms of bandwidth per carrier frequency, power and density without requiring a precision manufacturing process. Increased centre frequency accompanied by semiconductor technology scaling will result in the increase of wire density and throughput. In addition, frequency-independent loss-profile of the E-TUBE enables the adoption of advanced modulation schemes that improve the spectral efficiency without complex equalizers. This new interconnect scheme for next-generation high-speed input/output (I/O) interfaces would be attractive to parties interested in high throughput links, including but not limited to 100 G/400 Gbps chip-to-chip communications.

## Methods

### S-parameter measurement

The S-parameter measurement is performed using an Agilent N5227A Network Analyzer with Agilent N5250CX10 110-GHz Frequency Extender Modules. The signal is launched into the DUT via 1.00-mm connectors (Southwest Microwave, Inc.). Both ports of the Agilent N5250CX10s are connected to the input and the output ports of the DUT for two-port measurement. The transmission characteristic can be measured from 10 MHz to 110 GHz.

### Eye diagram and bit-error-rate measurement

To evaluate the performance of the interface, data transmission is demonstrated in the setup shown in Supplementary Fig. 3a–3c. An Anritsu MP1800A Pulse Pattern Generator is used for generating 2^31^–1 Pseudo Random Binary Sequence (PRBS). The baseband signal modulates the 73-GHz LO signal using a GaAs Schottky diode based balanced mixer (SAGE Millimeter Inc.). An Agilent E8257D Signal Generator with the S15MS WR15 Frequency Extension Module (OML Inc.) drives the LO port of the mixer for the generation of the 73-GHz sinusoidal LO signal. The modulated signal is transmitted through the DUT and then the signal received through the DUT is demodulated in the opposite way. Phase adjustment is performed at the LO port of the down-converting mixer for appropriate demodulation. Commercial mixers used for both up- and down-conversion show a 12-dB conversion loss, which is the main contributing factor degrading the signal-to-noise ratio (see Supplementary Fig. 3d,e).

## Additional Information

**How to cite this article**: Song, H. I. *et al.* Plastic straw: future of high-speed signaling. *Sci. Rep.* **5**, 16062; doi: 10.1038/srep16062 (2015).

## Supplementary Material

Supplementary Information
