# Supplementary material for: Plastic straw: future of high-speed signaling
Source: Sci Rep. 2015 Nov 3;5:16062. doi: 10.1038/srep16062 (PMC4630635; doi:10.1038/srep16062)
Supplement: Supplementary Information [file srep16062-s1.pdf]

Supplementary Information for

Plastic straw: future of high-speed signaling

Ha Il Song[1], Huxian Jin[1], and Hyeon-Min Bae[1]*

[1]Korea Advanced Institute of Science and Technology (KAIST), Department of Electrical Engineering, Daejeon, 305-701, Republic of Korea

*hmbae@kaist.ac.kr

Supplementary notes

1. Waveguide-length-dependent ripple in the S-parameter

A simplified two-port network model (Fig. 2a) of the proposed interconnect is created to analyze the ripple in S-parameter measurements. The E-TUBE has a symmetrical and bidirectional structure. Discontinuities occur at the boundaries between the microstrip line and the waveguide. For analysis, the discontinuities can be described by using scattering matrices where $r_i e^{j\alpha_i}$ and $t_i e^{j\beta_i}$ are reflection and transmission coefficients, respectively. The propagating wave can be expressed by the attenuation S along the waveguide, the propagation constant k at frequency ω, and the length of the waveguide l. Scattering matrices modeling the traveling waves in the waveguide and the microstrip are shown below.

$$\begin{bmatrix} u_1^- \\ w_1^+ \end{bmatrix} = \begin{bmatrix} r_1 e^{j\alpha_1} & t_2 e^{j\beta_2} \\ t_1 e^{j\beta_1} & r_2 e^{j\alpha_2} \end{bmatrix} \begin{bmatrix} u_1^+ \\ w_1^- \end{bmatrix} \tag{1}$$

$$\begin{bmatrix} w_2^- \\ u_2^+ \end{bmatrix} = \begin{bmatrix} r_2 e^{j\alpha_2} & t_1 e^{j\beta_1} \\ t_2 e^{j\beta_2} & r_1 e^{j\alpha_1} \end{bmatrix} \begin{bmatrix} w_2^+ \\ u_2^- \end{bmatrix} \tag{2}$$

$$\begin{bmatrix} w_2^{+} \\ w_1^{-} \end{bmatrix} = \begin{bmatrix} se^{-jkl} & 0 \\ 0 & se^{-jkl} \end{bmatrix} \begin{bmatrix} w_1^{+} \\ w_2^{-} \end{bmatrix} \tag{3}$$

$$\begin{bmatrix} u_1^{-} \\ u_2^{+} \end{bmatrix} = \begin{bmatrix} S_{11} & S_{12} \\ S_{21} & S_{22} \end{bmatrix} \begin{bmatrix} u_1^{+} \\ u_2^{-} \end{bmatrix} \tag{4}$$

The following equations express the transmission and reflection characteristics of the overall interconnect.

$$\left| S_{11} \right| = \left| \frac{ER_1 + E^{-1}s^2 R_2 (T_1 T_2 - R_1 R_2)}{E - E^{-1}s^2 {R_2}^2} \right|^2 \tag{5}$$

$$\left| S_{21} \right| = \left| s \frac{T_1 T_2}{E - E^{-1}s^2 {R_2}^2} \right|^2 \quad (T_i = t_i e^{j\beta_i},\ R_i = r_i e^{j\alpha_i},\ E = e^{jkl}) \tag{6}$$

$$\begin{aligned} &Group\ Delay = -\frac{d}{d\omega} \angle S_{21} \\ &\angle S_{21} = \tan^{-1}\left(\frac{\mathrm{Im}\, g\{sT_1T_2\}}{\mathrm{Re}\{sT_1T_2\}}\right) - \tan^{-1}\left(\frac{\mathrm{Im}\, g\{E - E^{-1}s^2 {R_2}^2\}}{\mathrm{Re}\{E - E^{-1}s^2 {R_2}^2\}}\right) \end{aligned} \tag{7}$$

Figure 2b shows the simulated results of Supplementary equations (S6) and (S7) under different lengths (e.g., 5 cm and 30 cm), where T_i, R_i, and s are frequency independent. Corresponding measurement results are provided to validate the modeling of the waveguide-length-dependent ripple. In this simulation, we intentionally created a waveguide model with high reflection at the discontinuities in order to emphasize the importance of careful ripple suppression. The ripples existing in the S-parameter and group delay plots increase in proportion to the length of the waveguide. The ripple deteriorates signal integrity and eventually increases the bit-error-rate.

Supplementary Figures S1a-S1c show the variation of the frequency-domain ripple with respect to various

parameters.

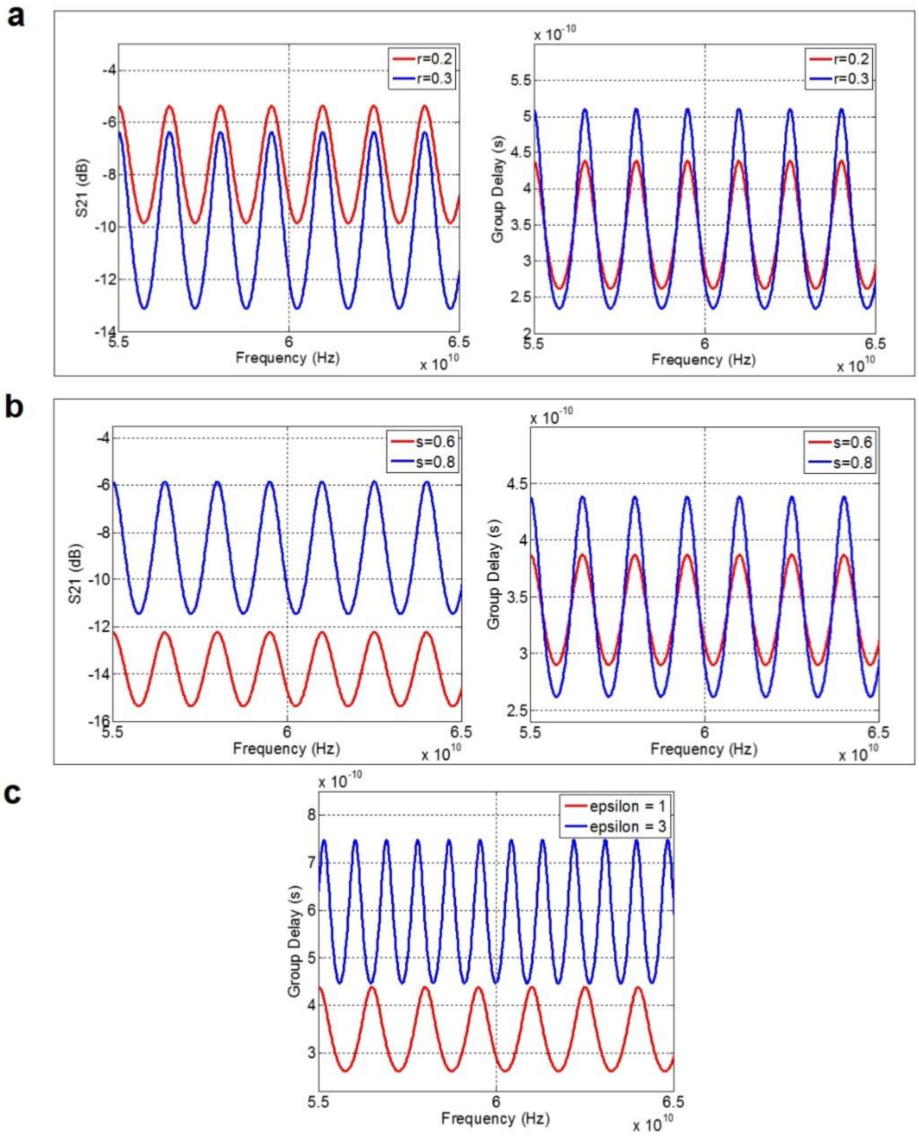

Supplementary Figure 1 | Analysis for ripple suppression. (**a**) The plots of S_{21} and group delay results according to reflection coefficient. (**b**) The plots of S_{21} and group delay according to the propagation loss. (**c**) A plot of group delay according to the dielectric constant of the waveguide.

As indicated in Supplementary Fig. S1a, we observe that the decreased reflection coefficient reduces the reflection at the boundary and decreases the magnitude of the ripple in S_{21} and group delay. Intuitively, a higher propagation loss suppresses the ripple in S_{21} and group delay as highly-attenuated reflected waves have a diminished adverse effect on the original waves (Supplementary Fig. S1b). However, the optimum channel loss should be determined carefully to strike a balance between the reflection and power budget of the link. Finally, the dielectric constant of

the waveguide should be made small (Supplementary Fig. S1c) as a large value of the dielectric constant ε_r increases the ripple in group delay. However, a small dielectric constant enlarges the dimension of the waveguide. Even though S_{21} is relevant to the dielectric constant of the waveguide, decreasing the dielectric constant does not suppress the frequency-domain ripple in S_{21}.

2. Broadband Microstrip-to-Waveguide Transition design

Each design parameter should be chosen carefully to maximize the bandwidth of the waveguide. Supplementary Figure S2 shows a simplified lossless lumped-circuit model of the waveguide and the interface.

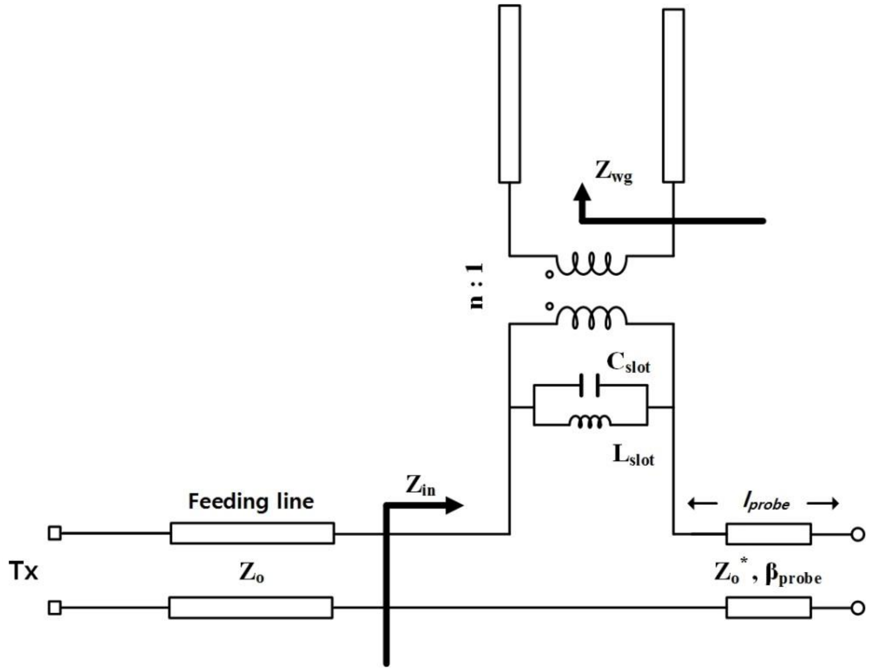

Supplementary Figure 2 | Simplified lossless lumped circuit model of the waveguide and the interface. The equivalent circuit model is composed of an LC resonator, an ideal transformer, and transmission lines. The inductance and the capacitance of the LC resonator should be determined appropriately to model the resonant frequency. The transformer in the model represents the energy transfer from the microstrip transmission line to the dielectric waveguide. In particular, n, the turns ratio of transformer, indicates the coupling efficiency between the mode of the microstrip line, quasi-TEM transmission mode, and that of the waveguide, TE fundamental transmission mode. The feeding line is followed by the open-circuited microstrip line called probe.

Under impedance matched condition,

$$Z_{in} = Z_0 \tag{8}$$

where input impedance is

$$Z_{in} = (n^2 Z_{wg} \parallel j\omega L_{slot} \parallel 1/\, j\omega C_{slot}) + (-jZ_0^{*} \cot(\beta_{probe} l_{probe}))\,. \tag{9}$$

Near resonance, $\omega = \omega_0 + \Delta\omega \approx \omega_0$, where the resonant frequency ω_0 is defined as $\sqrt{\frac{1}{L_{slot}C_{slot}}}$, and Supplementary equation (S9) can be rewritten for small $\Delta\omega$ as

$$\begin{aligned} Z_{in} &\simeq (n^2 Z_{wg} \parallel j\omega_0 L_{slot} \parallel 1/j\omega_0 C) + (-jZ_0^* \cot(\beta_{probe} l_{probe})) \\ &= \frac{n^2 Z_{wg} L_{slot}/C_{slot}}{L_{slot}/C_{slot} + jn^2 Z_{wg}(\omega_0 L_{slot} - \frac{1}{\omega_0 C_{slot}})} + (-jZ_0^* \cot(\beta_{probe} l_{probe})) \end{aligned} \tag{10}$$

The effect of loss can be accounted for by replacing the resonant frequency with a complex resonant frequency given by[1-2]

$$\omega_0 \leftarrow \omega_0 \left(1 + \frac{j}{2Q_0}\right) . \tag{11}$$

Now, the input impedance can be expressed as

$$Z_{in} = \frac{n^2 Z_{wg} L_{slot}/C_{slot}}{L_{slot}/C_{slot} + jn^2 Z_{wg}\left(\omega_0\left(1+\frac{j}{2Q_0}\right)L_{slot} - \frac{1}{\omega_0\left(1+\frac{j}{2Q_0}\right)C_{slot}}\right)} + (-jZ_0^* \cot(\beta_{probe} l_{probe})) \tag{12}$$

By substituting Supplementary equation (S12) for Supplementary equation (S8), we get

$$\frac{n^2 Z_{wg} \, {L_{slot}}/{C_{slot}}}{{L_{slot}}/{C_{slot}} + jn^2 Z_{wg}\left(\omega_0\left(1+\frac{j}{2Q_0}\right)L_{slot} - \frac{1}{\omega_0\left(1+\frac{j}{2Q_0}\right)C_{slot}}\right)} + (-jZ_0^{*}\cot(\beta_{probe} l_{probe})) \;=\; Z_0 \quad (13)$$

Supplementary equation (S13) can be rewritten using Q_0 assuming that

$(2Q_0)^2 = \left(2 \cdot \frac{resonance\ frequency}{bandwidth}\right)^2 \gg 1$ as given by

$$\left(4\frac{Z_0^{*}}{Z_0}\cot(\beta_{probe} l_{probe})\omega_0 L_{slot}\right)Q_0^{2} - \left(4\frac{Z_0^{*}}{Z_0}\cot(\beta_{probe} l_{probe})n^2 Z_{wg}\right)Q_0 + n^2 Z_{wg} = 0 \qquad (14)$$

Supplementary equation (S14) can be solved for Q_0 assuming $\cot(\beta_{probe} l_{probe}) \neq 0$, which is given by

$$Q_0 \simeq \frac{\frac{Z_0^{*}}{Z_0}\cot(\beta_{probe} l_{probe})n^2 Z_{wg} - \sqrt{\left(\frac{Z_0^{*}}{Z_0}\cot(\beta_{probe} l_{probe})n^2 Z_{wg}\right)^2 - \frac{Z_0^{*}}{Z_0}\cot(\beta_{probe} l_{probe})\omega_0 L_{slot} n^2 Z_{wg}}}{2\frac{Z_0^{*}}{Z_0}\cot(\beta_{probe} l_{probe})\omega_0 L_{slot}}$$

(15)

If $\cot(\beta_{probe} l_{probe})$ is zero, the quality factor becomes infinite.

The quality factor of the overall interface is given by

$$\frac{1}{Q_{eff}} = \frac{1}{Q_0} + \frac{1}{Q_c} + \frac{1}{Q_d} + \frac{1}{Q_{wg}} \approx \frac{1}{Q_0} \tag{16}$$

where Q_c, Q_d, Q_{wg} are the quality factors of conductor loss, dielectric loss, and waveguide loss. The quality factors of conductor and dielectric losses can be expressed simply as

$$Q_c = \frac{t}{\delta_c}\ ,\ Q_d = \frac{1}{\tan\delta}, \tag{17}$$

where t, δ_c, and $\tan\delta$ indicate the thickness of the conductor, the skin depth of the conductor, and the tangent loss of the substrate, respectively. The quality factor of the overall interface is governed by Q_0. Partial derivatives with respect to each parameter clarify the relations between the quality factor and corresponding parameters.

A parameter x defined as $\cot(\beta_{probe} l_{probe})$ affects the Q-factor as given by

$$\frac{\partial Q_{eff}}{\partial x} = \frac{-\left(\frac{Z_0^*}{Z_0}\omega_0 L_{slot}\right)^2 n^2 Z_{wg} x}{\left(2\frac{Z_0^*}{Z_0}\omega_0 L_{slot} x\right)^2 \sqrt{\left(\frac{Z_0^*}{Z_0} n^2 Z_{wg} x\right)^2 - \frac{Z_0^*}{Z_0}\omega_0 L_{slot} n^2 Z_{wg}}} = \frac{-P_1}{Q_1\sqrt{R_1}} x \tag{18}$$

where P_1, Q_1 are positive and R_1 is a real value.

If x is greater than zero, increasing x lowers the quality factor. Otherwise, if x is less than zero, decreasing x lowers the quality factor. The partial derivative with respect to x shows that the bandwidth can be expanded when

χ is made positive infinite or negative infinite. Indeed, χ can be made positive/negative infinite by adjusting the length of probe to be equal to the half wavelength at resonant frequency.

Provided that $\cot(\beta_{probe} l_{probe})$ is equal to positive or negative infinite, Supplementary equation (S15) can be simplified as

$$Q_{eff} \simeq \frac{n^2 Z_{wg}}{\omega_0 L_{slot}} \tag{19}$$

The partial derivative with respect to resonant frequency ω_0 is negative, indicating that the bandwidth broadens with increasing ω_0.

$$\frac{\partial Q_{eff}}{\partial \omega_0} = \frac{-n^2 Z_{wg}}{\omega_0{}^2 L_{slot}} < 0 \tag{20}$$

The relations between the quality factor and the coupling coefficient n^2 can be derived in a similar manner.

$$\frac{\partial Q_{eff}}{\partial n^2} = \frac{Z_{wg}}{\omega_0 L_{slot}} > 0 \tag{21}$$

The partial derivative with respect to the coupling coefficient n^2 is positive. This indicates that a lower coupling coefficient increases the bandwidth. The excitation of the surface wave under a thick substrate at millimeter wave frequencies is not negligible. A thick and high-ε_r substrate leads to a low coupling coefficient, which, in turn, increases the bandwidth. Two core substrates are made of 0.254-mm thick Rogers RO3010 ($\varepsilon_r = 10.2 @ 10GHz$).

Consequently, the carrier frequency, substrate thickness, dielectric constant, and probe length should be determined appropriately to achieve a wide bandwidth.

3. **Eye diagram measurement**

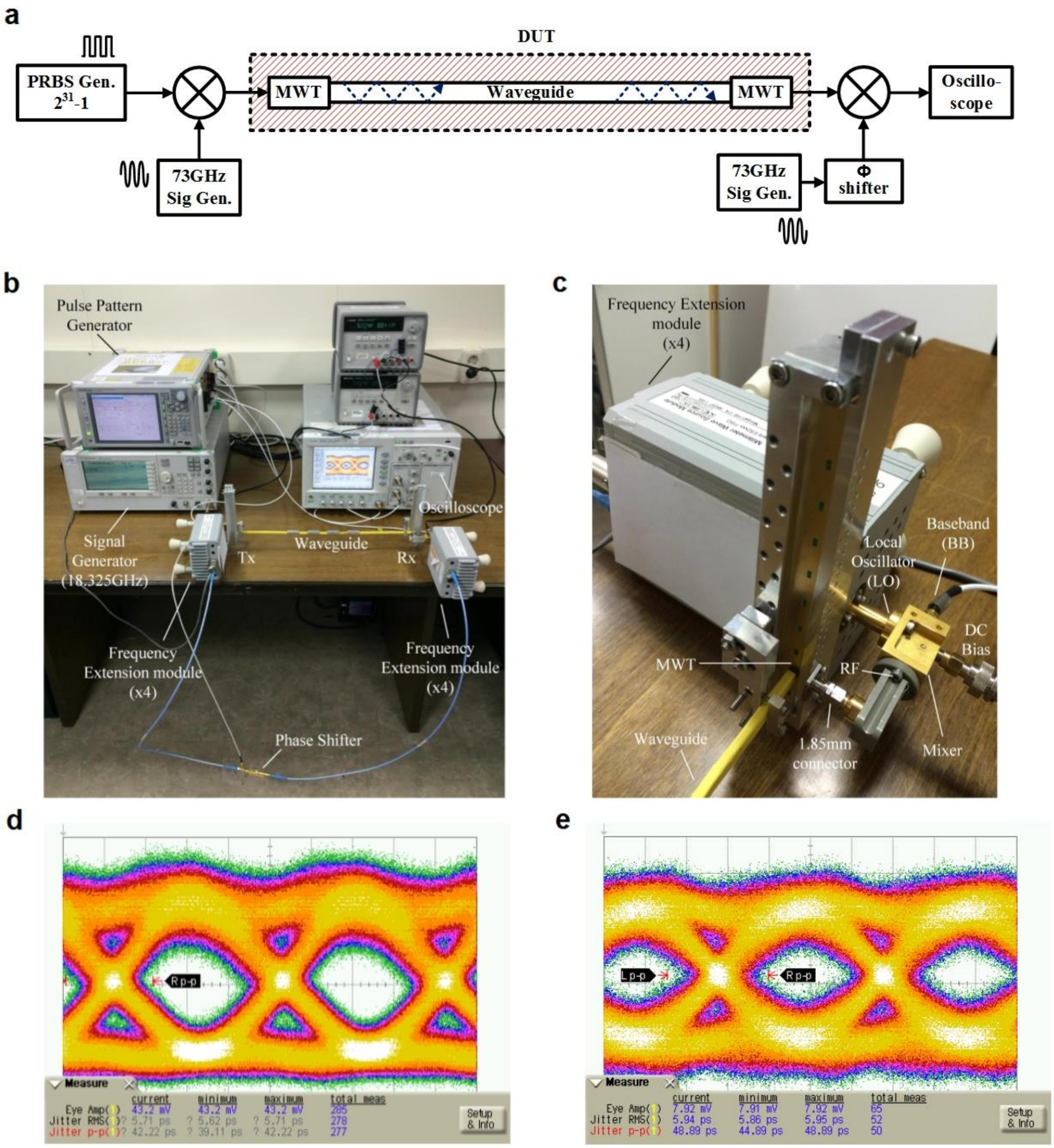

Supplementary Figure 3 | (**a**) A schematic diagram of eye diagram measurement setup. (**b**),(**c**), Photographs of eye diagram measurement setup (**d**) Eye diagram at 12.5 Gb/s without the DUT. (e) Eye diagram at 12.5 Gb/s in connection with the DUT.

4. The Microstrip-to-Waveguide Transition in the E-TUBE interface

Compared to the other broadband dielectric waveguide solutions, the proposed transition scheme couples the waveguide vertically to the board, which achieves high area efficiency. In addition, the implementation of the transition by using a standard PCB lamination process reduces the manufacturing cost of a connector significantly.

On the other hand, compared to the conventional microstrip-to-waveguide transitions, the proposed transition achieves an unprecedented level of bandwidth per carrier frequency thanks to the low-Q design. Moreover, we demonstrate for the first time, a single-sideband transmission through the waveguide, which is achieved by optimized coupling between the microstrip line and the waveguide. Supplementary Figure S4 shows a slot coupled Microstrip-to-Waveguide Transition (MWT). The MWT is designed to suppress the reflection occurring at the discontinuities between the waveguide and the microstrip line. The quasi-TEM transmission mode along the microstrip is coupled to TE_{10} fundamental transmission mode in the waveguide.

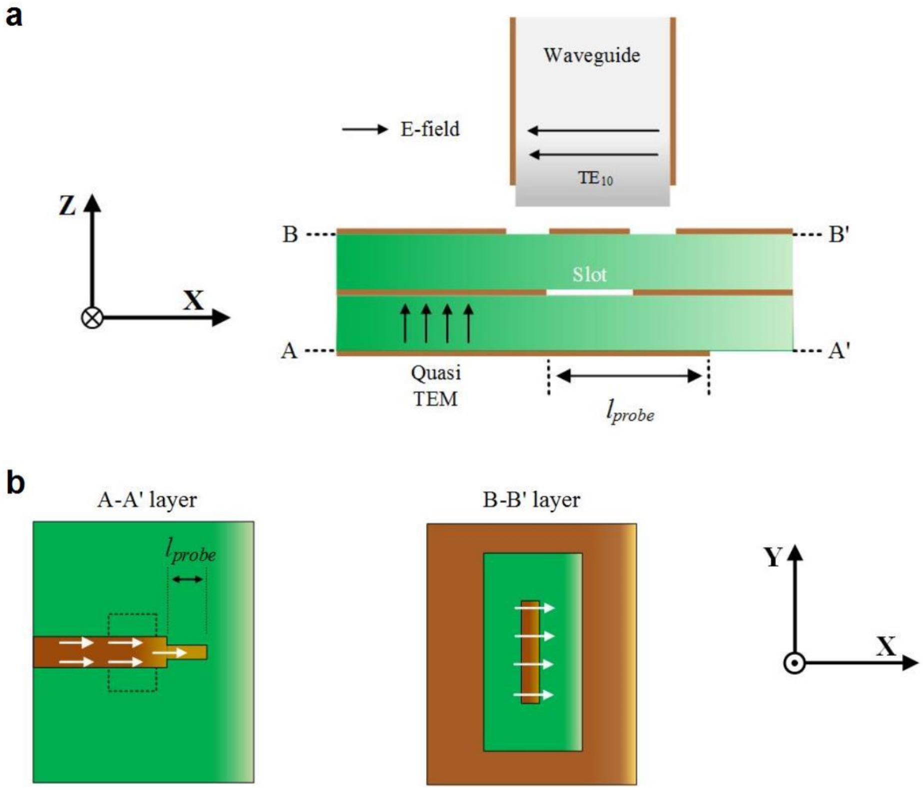

Supplementary Figure 4 | A cross-section of a Microstrip-to-Waveguide Transition. (**a**) The signal is transferred from the microstrip line on the bottom plane (A-A' layer) to the patch on the top plane (B-B' layer) in the transition. The radiated magnetic field from the current on the patch couples to the fundamental mode of the waveguide. (**b**) A bottom (A-A' layer) and a top (B-B' layer) plane view of the transition. An open circuited

narrow microstrip line extending the feeding line is referred to as a probe. The current on the microstrip line couples to the patch by inducing a current.

An open-circuited narrow microstrip line extending the feeding line, referred to as a probe, is critical for the frequency response of the MWT. The length of the probe, l_{probe}, significantly affects the roll-off response at the cutoff frequency since the coupling between the microstrip and the waveguide transmission modes is sensitive to the XZ plane (E plane) dimensions of the MWT. The electrical current, flowing through the microstrip line, couples to the patch by inducing a current in the X direction (Supplementary Fig. S4b) and the radiated magnetic field from the induced current on the patch couples to the TE_{10} mode of the waveguide.

The amount of the signal power that is not coupled to the waveguide is regarded as a coupling loss. Uncoupled signal is either radiated to the free space in backward direction or dissipated in the forms of dielectric and conductor losses.

Supplementary Figure S5 shows the measured S_{21} response with respect to the length of probe, l_{probe}. The red line shows the roll-off of 7.21dB/GHz when the length of probe is set at the optimum value L_{opt}. The blue and green lines show the roll-offs of 4.57 dB/GHz and 3.46 dB/GHz when the probe lengths are $(L_{opt} + 0.2)$ mm and $(L_{opt} - 0.2)$ mm, respectively. The sharpest roll-off response is achieved when $l_{probe} = L_{opt}$ = 0.7 mm.

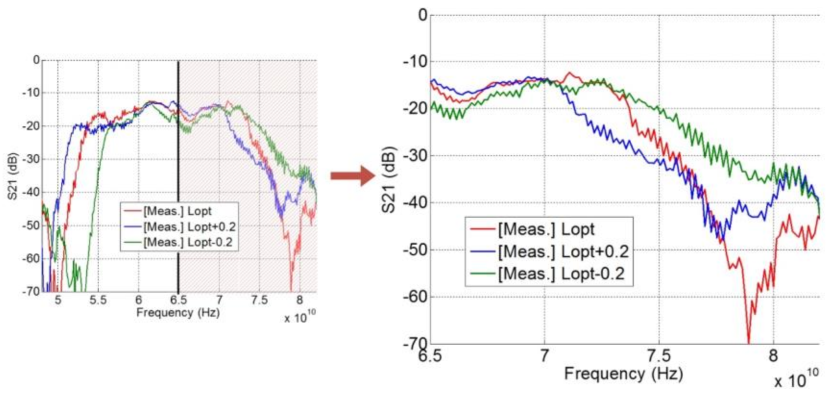

Supplementary Figure 5 | A graph of measured channel response while varying the length of the probe. The roll-off at the upper corner frequency is maximized when the length of l_{probe} is L_{opt}.

As a result, l_{probe} is critical for achieving sharp roll-off response at the upper corner frequency, which enables single sideband transmission without any additional filtering process.

5. The equalization scheme for the E-TUBE link

The frequency dependent loss of a 0.5-m waveguide itself is less than 1 dB over the passband. However, the coupling loss occurring at the MWT causes nearly 5 dB variation over the passband. It is because the broadband coupling between the microstrip and the waveguide transmission modes is enabled by generating multiple resonances in the MWT.

Both magnitude and group delay variations of a channel cause inter-symbol interference (ISI) and degrades the bit-error-rate performance of a link. In order to characterize the ISI of the E-TUBE link, a pulse response is investigated as shown in Supplementary Fig. S6 (1 UI corresponds to 50 ps). A 73.3-GHz carrier signal is modulated with a 50-ps pulse and transmitted through the E-TUBE link. The demodulated pulse response shows that both pre- and post-cursor ISIs are created.

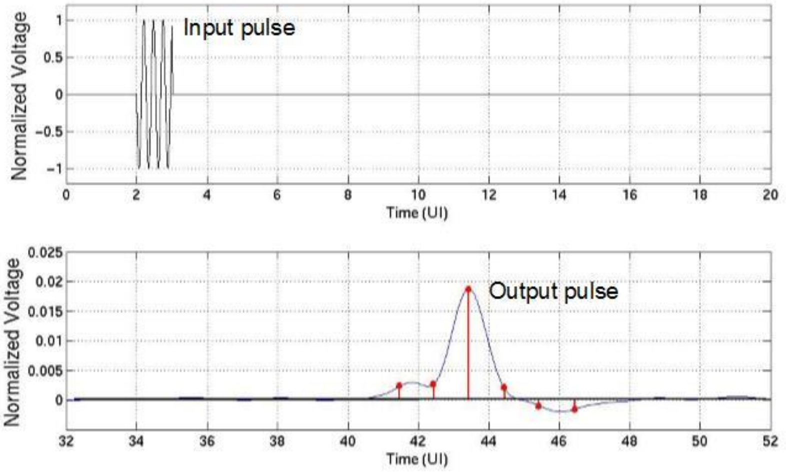

Supplementary Figure 6 | The pulse response of the E-TUBE channel. The input signal is modulated pulse whose width is 50 ps and carrier frequency is 73.3 GHz. The demodulated output is shown in the bottom.

Supplementary Figure S7 shows the block diagram of a tentative three-tap pre-emphasis finite-impulse-response filter. The emphasized pulse reduces the pre-and the post-cursor ISI in the receiver side.

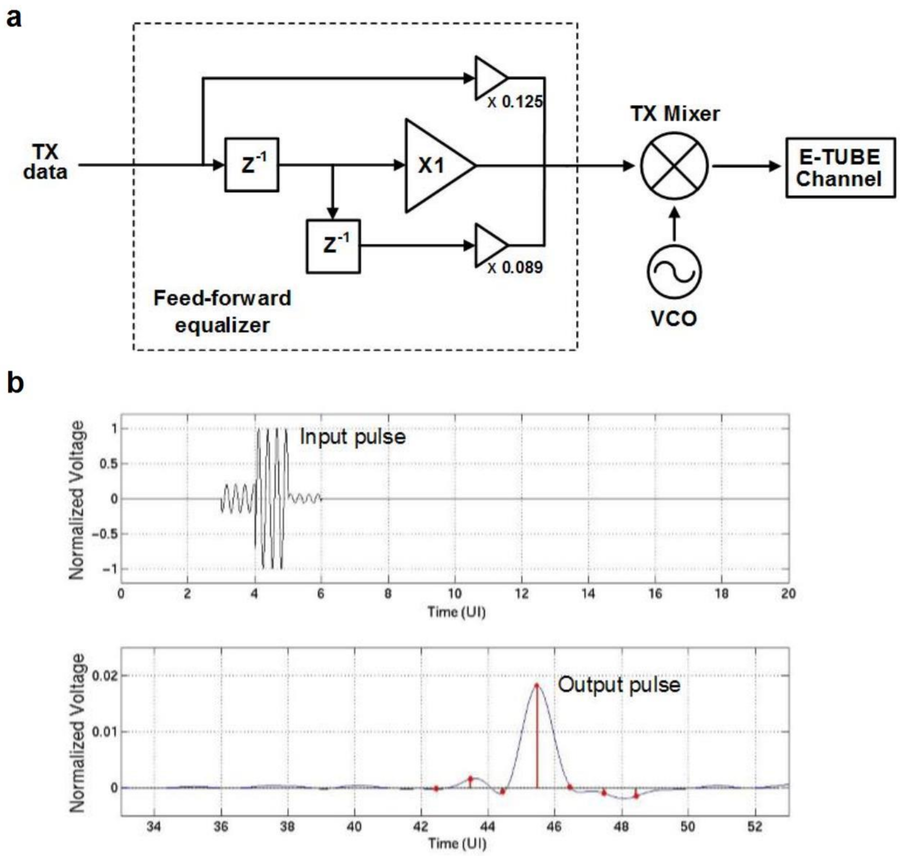

Supplementary Figure 7 | (a) A pre-emphasis digital filter with a pre-tap and a post-tap. The emphasized pulse modulates the 73.3 GHz sinusoidal carrier signal and the modulated signal transmits to the E-TUBE channel. (**b**) The input signal is modulated pulse with a pre-cursor and a post-cursor.

A two-pre-tap-and-three-post-tap scheme further reduces the ISI of the channel (Supplementary Fig. S8).

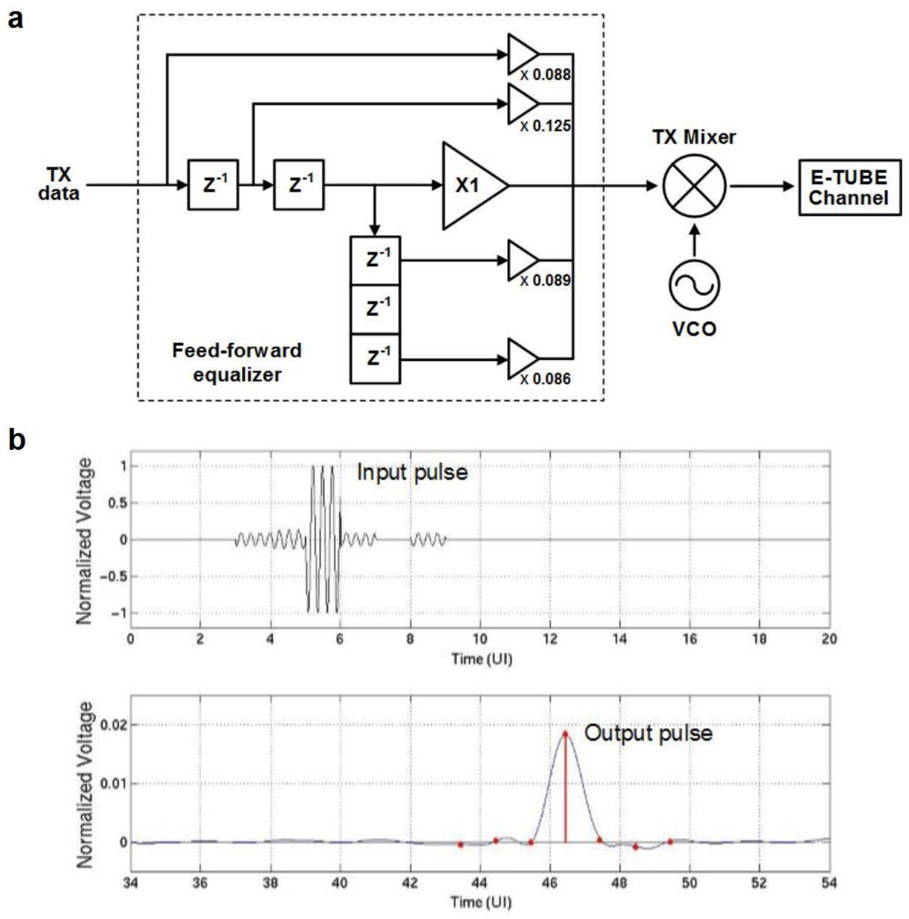

Supplementary Figure 8 | (**a**) A pre-emphasis digital filter with additional 2nd pre-tap and 3rd post-tap. (**b**) The input signal is modulated pulse with additional pre-cursor and post-cursor.

Supplementary Figure S9 shows the eye diagrams of the E-TUBE link with different pre-emphasis schemes. It is clear that conventional TX pre-emphasis scheme is effective for ISI cancellation of the E-TUBE link. Because the reflection occurring at the MWT dominantly causes the ISI of the E-TUBE link, the length of the E-TUBE has negligible impact on the ISI. Therefore, a TX-side equalization scheme is appropriate for the E-TUBE link.

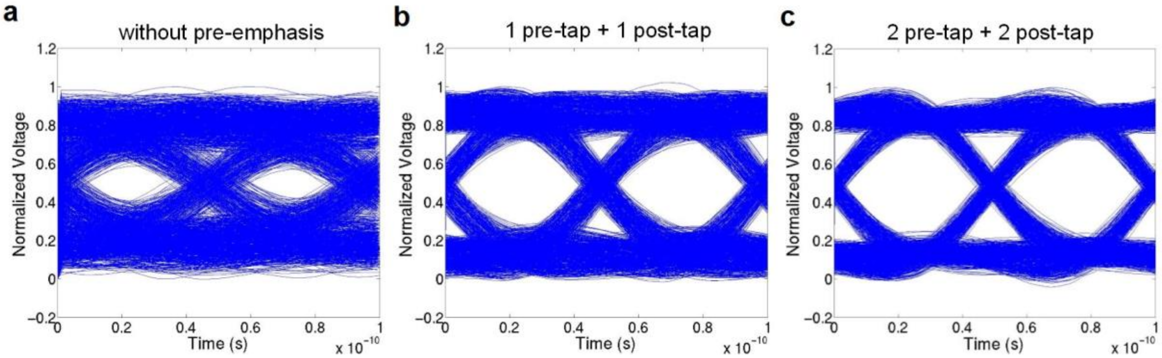

Supplementary Figure 9 | Eye diagrams at 20 Gb/s with PRBS length of 2^{14}-1. (**a**) without pre-emphasis (**b**) with 1 pre-tap and 1 post-tap (**c**) with 2 pre-tap and 2 post-tap.

6. Manufacturing and alignment tolerances

Manufacturing tolerance of the interface can be estimated through simulations while varying the thickness of the materials used for the laminated printed circuit board (PCB) and the horizontal dimensions of the waveguide. In the PCB lamination process, a stack of multiple layers is bonded together using a prepreg, while applying heat and pressure. A sheet of prepreg with the thickness of 60 um was used, where the thickness can vary as much as 20 % due to process variations. Supplementary Figure S10 shows the simulated S_{21} while varying the thickness of the prepreg by +/- 20%.

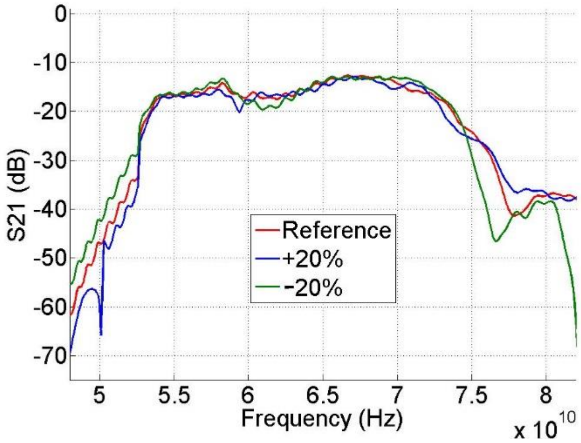

Supplementary Figure 10 | Characterization of manufacturing tolerance. The simulated results for thickness variation of the PCB.

We have observed S_{21} degradation of maximum 2 dB as shown in Supplementary Fig. S10 and we found that 2 dB

fluctuation has negligible impact on the overall performance. As such, the E-TUBE interface can be mass produced without any modification to the current PCB lamination process.

In addition, the manufacturing tolerance of the E-TUBE itself is estimated via simulations. The rectangular cross-section of the waveguide (width: 4 mm, thickness: 2 mm) is given variations as much as 10 % percent while monitoring S_{21}.

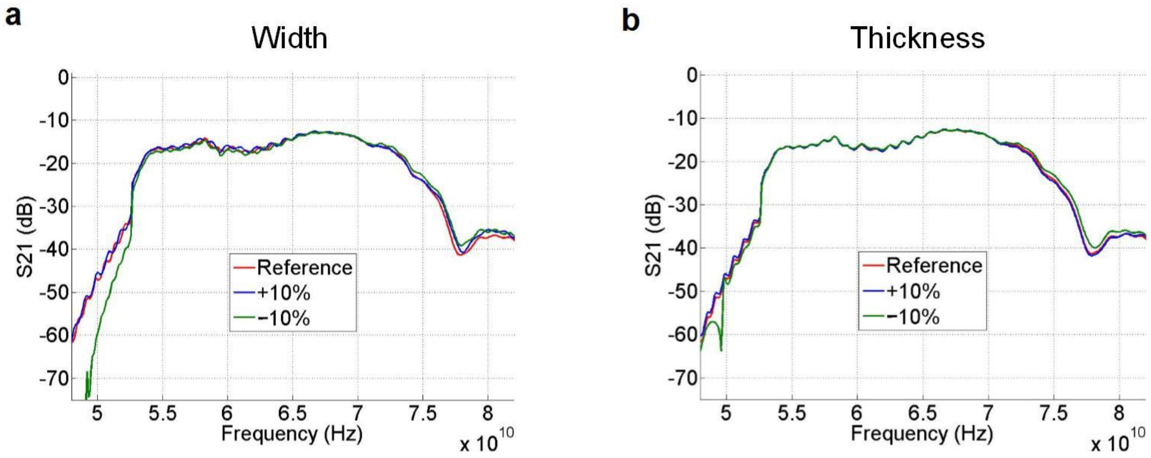

Supplementary Figure 11 | Characterization of manufacturing tolerance. (**a**) The simulated results for the width variation of the waveguide. (**b**) The simulated results for the thickness variation of the waveguide.

It is found that 10% variations in the width and thickness result in S_{21} degradation of maximum 1dB, as shown in Fig. S11. 1-dB degradation has negligible impact on the E-TUBE performance.

Alignment tolerance is also estimated by varying the gap between the waveguide and the board.

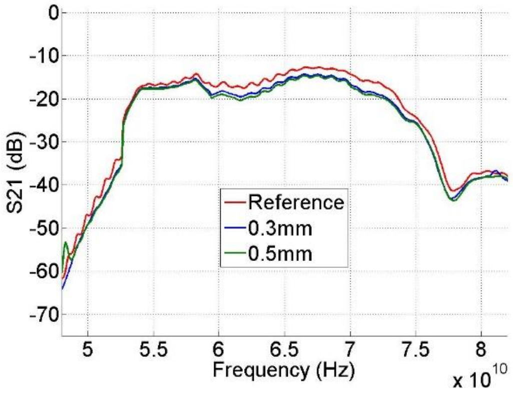

Supplementary Figure 12 | Characterization of Alignment tolerance. The simulated result with a gap of 0.3 mm and 0.5 mm between the waveguide and the board.

The S_{21} degradation caused by 0.3-mm airgap is less than 2 dB over the passband and additional 1dB degradation is observed when the airgap increases to 0.5 mm as shown in Supplementary Fig. S12.

The effects of vertical and horizontal misalignment of the waveguide are shown in Fig. 4a in the manuscript.

7. A comparison table with other dielectric waveguide solutions.

	[3]	[4]	[5]	This work
Distance (m)	0.12 / 1	0.6 / 1	2 / 7.6	0.5
Bandwidth / Carrier Frequency	0.16 / 0.05	0.08 / 0.06	0.1 / 0.06	0.27 (single sideband transmission)
Channel loss	N/A	N/A	Coupling loss : 6dB Waveguide loss: 1.5dB/m	Coupling loss : 5.5dB Waveguide loss: 4dB/m
Bending radius (mm)	255	N/A	N/A	4.5
Antenna	On-board antenna	On-chip bondwire antenna	On-chip bondwire antenna	On-board transition
Waveguide	Dielectric waveguide (polystyrene)	Dielectric waveguide (polypropylene)	Dielectric waveguide (teflon)	Metal clad dielectric waveguide (polyethylene foam)

Supplementary Table 1 | A comparison table with other dielectric waveguide solutions.

Supplementary references

1. D. M. Pozar. *Microwave engineering 4 edn*, (Wiley, New York, 2009), 272-316
2. Li, Long, and C. H. Liang. Analysis of resonance and quality factor of antenna and scattering systems using complex frequency method combined with model-based parameter estimation. *PROG ELECTROMAGN RES* **46,** 165-188 (2004).
3. Fukuda, S. et al. A 12.5+12.5 Gb/s Full-Duplex Plastic Waveguide Interconnect. *IEEE J. Solid-State circuits.* **46,** 3113-3125 (2011)
4. Tytgat, M. & Reynaert, P. A plastic waveguide receiver in 40nm CMOS with on-chip bondwire antenna. *Proc, of ESSCIRC. 2013*, 335-338 (2013)
5. Kim, Y. et al. High Speed mm-Wave Data-Link Based on Hollow Plastic Cable and CMOS Transceiver. *IEEE Microw. Compon.Lett.* **23,** 674-676 (2013)
